# 

**Published:** 1908-01

**Authors:** 


					﻿'ypuixxni.
JANUARY, 1908.
No. 7.
TEXAS
MEDICAL
JOURNAL
PUBLISHED AT AUSTIN, TEXAS, BY
t\ E. DANIEL, M. D.,	-	- Proprietor
PRINTED BY THE VON BOECKMANN-JONES CO.
t Subscription, $l.OO a_Year, in Advance. •	.	. Single Copies, 10 Cents
Entered at the Postoffice at Austin, Texas, as Second-class Mail Matter
				

